# “Sometimes it is difficult for us to stand up and change this”: an analysis of power within priority-setting for health following devolution in Kenya

**DOI:** 10.1186/s12913-018-3706-5

**Published:** 2018-11-29

**Authors:** Rosalind McCollum, Miriam Taegtmeyer, Lilian Otiso, Nelly Muturi, Edwine Barasa, Sassy Molyneux, Tim Martineau, Sally Theobald

**Affiliations:** 1 Department of International Public Health, Liverpool School of Tropical Medicine, Liverpool, UK; 2 LVCT Health, Nairobi, Kenya; 3 Health Economics Research Unit, KEMRI-Wellcome Trust Research Programme, P.O. Box 43640-00100, Nairobi, Kenya; 4 Nuffield Department of Medicine, University of Oxford, Oxford, UK; 5 KEMRI Centre for Geographic Medicine Research – Coast, and Wellcome Trust Research Programme, Nairobi, Kenya; 6 Centre for Tropical Medicine, University of Oxford, Oxford, UK

**Keywords:** Power, Priority-setting, Kenya, Devolution

## Abstract

**Background:**

Practices of power lie at the heart of policy processes. In both devolution and priority-setting, actors seek to exert power through influence and control over material, human, intellectual and financial resources. Priority-setting arises as a consequence of the needs and demand exceeding the resources available, requiring some means of choosing between competing demands. This paper examines the use of power within priority-setting processes for healthcare resources at sub-national level, following devolution in Kenya.

**Methods:**

We interviewed 14 national level key informants and 255 purposively selected respondents from across the health system in ten counties. These qualitative data were supplemented by 14 focus group discussions (FGD) involving 146 community members in two counties. We conducted a power analysis using Gaventa’s power cube and Veneklasen’s expressions of power to interpret our findings.

**Results:**

We found Kenya’s transition towards devolution is transforming the former centralised balance of power, leading to greater ability for influence at the county level, reduced power at national and sub-county (district) levels, and limited change at community level. Within these changing power structures, politicians are felt to play a greater role in priority-setting for health. The interfaces and tensions between politicians, health service providers and the community has at times been felt to undermine health related technical priorities. Underlying social structures and discriminatory practices generally continue unchanged, leading to the continued exclusion of the most vulnerable from priority-setting processes.

**Conclusions:**

Power analysis of priority-setting at county level after devolution in Kenya highlights the need for stronger institutional structures, processes and norms to reduce the power imbalances between decision-making actors and to enable community participation.

**Electronic supplementary material:**

The online version of this article (10.1186/s12913-018-3706-5) contains supplementary material, which is available to authorized users.

## Background

Practices of power lie at the heart of policy processes [1]. In both devolution and priority-setting, actors seek to exert power through influence and control over material, human, intellectual and financial resources [2, 3]. In both processes, a range of actors, each with their own values, needs and interests must make judgements and decisions about the selection of priorities contained within plans and budgets. Priority-setting and devolution can be viewed as two sides to the same coin. Potential threats to priority-setting will also threaten the potential success of devolution.

Priority-setting, the distribution of resources among competing healthcare services, patients or patient groups, arises as a consequence of the needs and demand for healthcare resources (such as budget, staff time, equipment and facilities) exceeding the resources available [4–6]. As a result, some means of choosing between competing demands is required [6]. Ideally, priority-setting is explicit, seeking to set clear priorities, with a transparent rationale and resource allocation based on agreed upon criteria [7]. Some of these criteria include “benefit, evidence, cost, efficiency, equity, equality, benefit to a country’s economy, severity of disease, prevalence of disease, solidarity, protection of the vulnerable, and more” (p21 [8]). While sometimes viewed as a purely technical process, priority setting is typically a complex, value laden process where actor’s values and interests are brought to bear, negotiating decisions about which values or principles should dominate as political, institutional and managerial factors come into play [9].

Decentralisation is a dynamic process which transfers authorities or powers for decision-making, planning and management of public services from national to sub-national levels [10]. Decentralisation reforms may be classified as four main types: de-concentration occurs when authority for administrative functions shifts to sub-national offices within the Ministry of Health; delegation occurs when semi-autonomous agencies are granted new powers (typically still administrative); devolution occurs when administrative, political and fiscal responsibilities shift to the sub-national level of locally elected government, and privatisation when ownership is granted to private bodies [11, 12].

Devolution reforms most accurately describe the transition which began in 2013 in Kenya. Devolution ought to transform authority, power and responsibility for implementation between actors. Despite their popularity, devolution reforms have a mixed record in terms of realising their many objectives, such as improving national unity, equity, efficiency, responsiveness, accountability and community participation [11, 12]. This is often due to difference between the official public policy goals for health and the goals of individuals, including local politicians and other decision-making actors [13].

The success (or failure) of both priority-setting and devolution are heavily dependent on how actors respond to changing distributions of power. Power is “the degree of control over material, human, intellectual and financial resources exercised by different sections of society” (page41 [2]). Power is both dynamic and relational, rather than absolute and is exercised through the social, economic and political relations between individuals and groups [2]. The distribution of power can change with the context, circumstances and interest of actors and can be expressed in a range of forms from domination and resistance to collaboration and transformation [2].

**Fig. 1:**
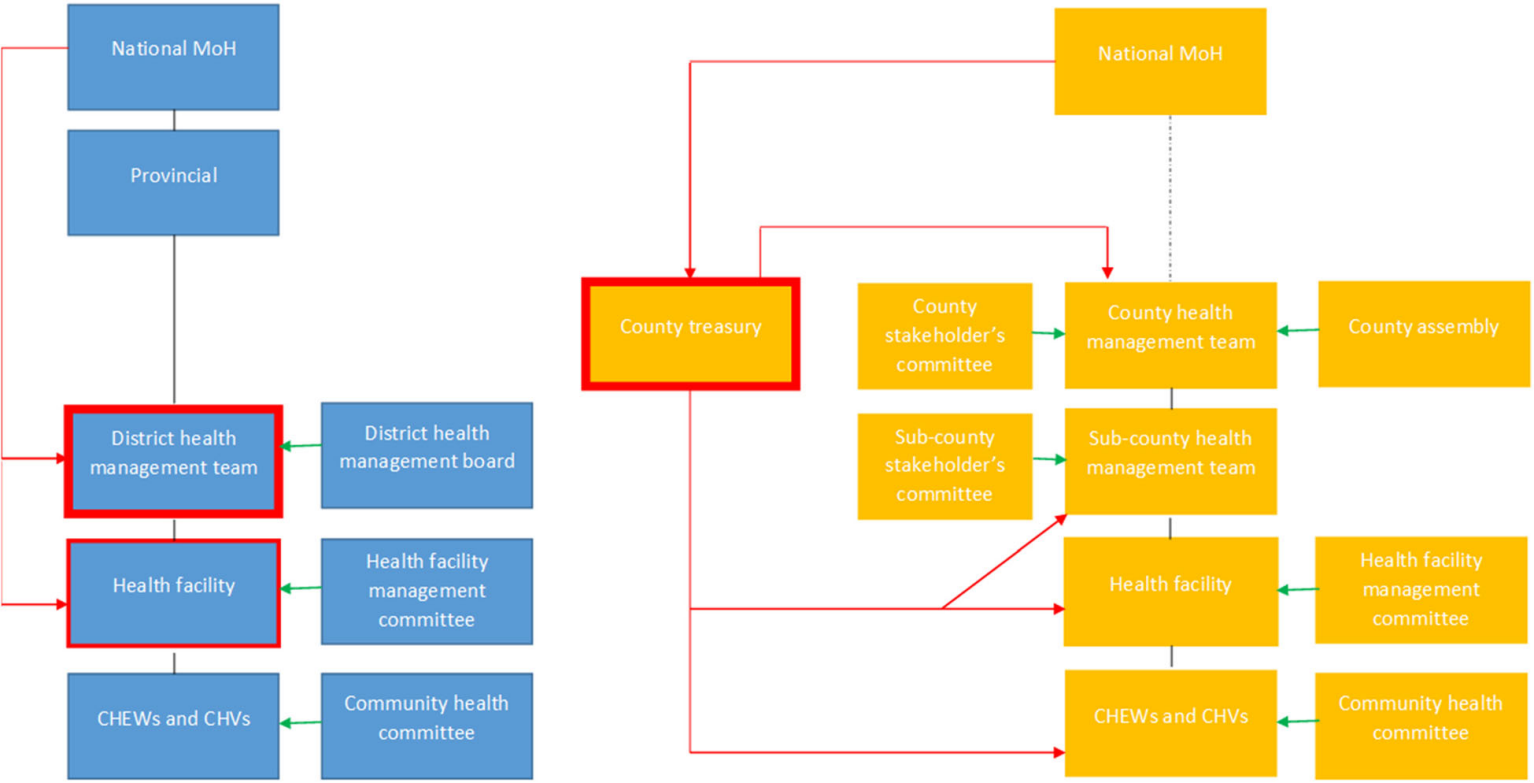
Pre (left) and post (right) devolution health systems structures in Kenya. Green arrows indicate governance, black lines indicate supervision pathways, red arrows indicate flow of funding. The black dotted line between national and county indicates the new relationship between national and county governments. Red box surrounding some boxes indicates structures which receive funding directly from national level

In 2013 Kenya’s governance system was transformed, following the ambitious devolution of political, fiscal and administrative functions to 47 new sub-national county governments [14]. This led to revised roles for national Ministry of Health and increasing responsibilities now held at the newly formed County government level. Devolution reform objectives in Kenya seek to “tackle long- term, deeply entrenched disparities between regions; increase the responsiveness and accountability of government to citizens; allow greater autonomy to different regions and groups, and re-balance power away from a historically strong central government” (page2 [15]). This led to changing roles and power for actors across the health system (see Table 2 [16]). Roles were reduced for national level in coordinating partners, recruitment and management of health workers and planning for budget allocation. National level actors have retained responsibility for policy development, quality assurance and provision of national referral (level four) services. The former district level (now largely considered similar to the sub-county level) continues to hold responsibility to implement and deliver health services, but no longer has control over budget allocation (compared with prior to devolution when annual planning and budget allocation responsibilities for management of user fees had been decentralised to this level [17, 18]) (see Fig. 1). Prior to the introduction of devolution reforms in 2013 Kenya had a ‘highly decentralised form of de-concentration’ with hospitals experiencing some degree of autonomy over management functions [19]. Following devolution, previous studies have found a recentralisation of management (including financial management) from health facility to county level, particularly within county hospitals [17, 19]. The changing roles and responsibilities for national, county, district/sub-county and community level before and after devolution have been summarised elsewhere (see Table 2 [16]).

**Fig. 2:**
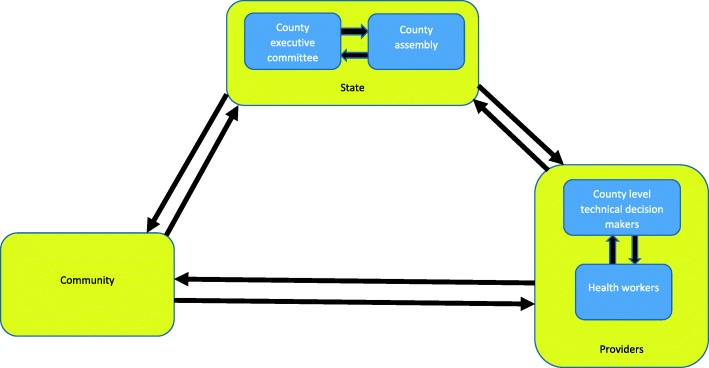
Power interfaces between decision-making actors (modified Brinkerhoff and Bossert, 2008)

Kenya’s health system is funded from four main sources – the government from taxes and donor funding at both national and county levels; off-budget donor funding; National Hospital Insurance Fund member contributions; private health insurance member contributions, and out of pocket spending at point of care [20]. This paper will examine the power dynamics relating to budget allocation and annual planning responsibilities for actors at the county level, who also hold responsibility for developing the five year county integrated development plan, for health service delivery for level one to four services (community, primary and county referral services), for recruitment and management of health workers and for coordination of partners. Funds available at county level come from three possible sources; 1) transfers from central government which comprise an equitable share allocated to all the 47 counties from national general revenue collections using a revenue allocation formula, conditional grants ring fenced for specific functions, and an equalization fund for the 14 previously marginalized counties, 2) locally generated revenue, and 3) donor funding (see Table 1 [21]). The annual planning and budgeting cycle has been described elsewhere (Fig. 2 [16]). In summary, national circulars are issued, which prompt the development of the annual plan and budgeting process. As part of this process, the county budget steering committee and county executive committee set budget ceilings and develop the county fiscal strategy paper; public participation meetings should be held for the identification and validation of priorities set, with the health department working to align their plan and budget to meet budget ceilings assigned, under the oversight of the county assembly prior to finalisation and approval from both the county assembly and the county executive committee [16]. County level actors engaged in the priority-setting process for the annual budget planning cycle (see Fig. 2, [16])include:

- State actors, including governor and members of county executive committee (CEC) (representatives appointed by the governor for ten service departments within the county, including the county executive member for health, who may or may not have health experience) and members of county assembly (elected local ward representatives)
- Health service providers, including county level technical decision makers (e.g. members of county health management team) and health actors at other levels, including sub-county health management team and health workers at facility and community levels
- Community members, including selected community representatives who sit on specific quarterly health planning and review committees along with the general community, who should be engaged in priority-setting through public participation forums.

**Table 1:** Dimensions of Gaventa’s power cube (Barasa et al. 2016; Rowlands 1997; Veneklasen et al. 2002)

Spaces for power	
Closed spaces	Elites make decisions behind closed doors, which in Kenya include discussions between county executive committee members and the governor or among members of county assembly;
Invited spaces	Citizens are invited to participate by authorities, which in Kenya includes the public participation forums;
Claimed spaces	Less powerful actors claim spaces from the power holders, which in Kenya includes the use of social media platforms by citizens to raise awareness about poor quality services.
Places for power	
Global	Globalisation shifts traditional understandings of the location and exercise of power. Global actors, forces and structures in turn influence and shape national and local level power relationships. In Kenya this includes international pressure for devolution.
National County Health worker	The role of the national level in power dynamics, influences the legitimacy and power dynamics at other (sub-national) and local levels. Decentralisation, transforms national and local power relationships. In Kenya, this has contributed to reduced power for national level to identify, plan and budget for health priorities and actions at sub-national levels, with greater power now held at county level and lower levels of power held by health workers at sub-county and health facility level compared with prior to devolution.
Local	Local levels may be dependent on other levels for the extent to which power is legitimated. In Kenya, community level actors may be involved with priority-setting through public participation forums, or through community representation in other existing committees at community and facility levels.
Forms and visibility of power	
Visible power’	Observable decision making. This includes the visible and definable rules, structures, authorities, institutions and procedures for decision-making. Strategies targeting this level seek to change the ‘who, how and what’ to increase accountability of priority-setting processes.
‘Hidden power’	Setting the political agenda, is less obvious. Certain powerful people and institutions maintain their influence by controlling who is involved with decision-making and what is on the agenda. Actions to address this level include empowering advocacy strategies that seek to strengthen organisations of poor and marginalised people to influence the way in which political agenda is shaped.
‘Invisible power’	Shaping meaning and what is important. Problems and issues are kept from the minds of the actors involved, by influencing how they think about their place in the world and controlling access to information, so that people are unable to make informed choices. In this dimension power operates at a deeper ‘invisible’ level, so that actors may unwittingly follow against their own best interests, thereby avoiding conflict by making it impossible for people to imagine anything different to the status quo. Power is closely associated with ideology. Beliefs, values, attitudes and ways of analysing life, enforced by structures such as family, education system, religion, the media, the economy and the state, tend to reinforce the dominant ideology and power of the dominant groups within it. Change strategies at this level target social and political culture and individual consciousness.

In the wake of devolution’s reforms in Kenya we are left with a number of questions, relating to the health sector. How is power re-distributed? And how does this vary across counties? Who benefits from this re-distribution? How are the new powers used? Do new priorities lead to improved health equity? We aim to provide a power analysis of priority-setting at the new county level, to understand how power influences priority-setting in Kenya.

## Methods

### Theory and practice

This paper presents the analysis of data collected as part of a wider study which sought to explore priority-setting for community health and equity since devolution. We used a naturalistic approach to unpack power in its fluid and multiple forms and to observe the changes for priority-setting following devolution as they occurred [22, 23]. We used multiple qualitative methods including key informant interviews, in-depth interviews and focus group discussions, including a range of perspectives from national to community level.

**Fig. 3:**
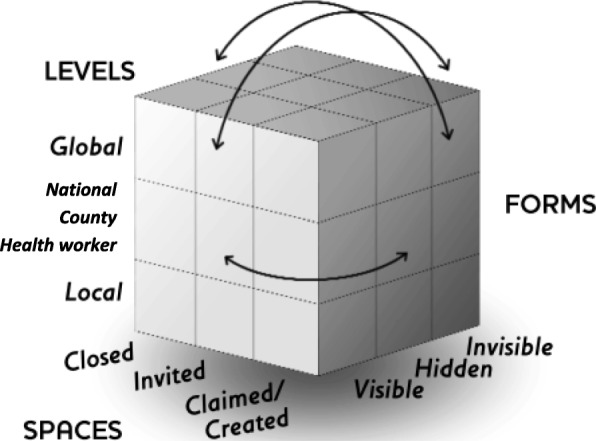
Gaventa’s power cube (2006)

To explore power dynamics within priority-setting for health, we adopted two complementary frameworks as the basis for a power analysis - Gaventa’s power cube (2006) [24] (see Fig. 3), incorporating aspects of Veneklasen’s expressions of power (2002) [2] to more fully explore power and the complexity of the social, political and economic factors (among others) which influence it. The power cube considers the spaces, places and forms of power (see Table 1). Spaces for power include opportunities and channels where actors can potentially influence policy and decisions [24]. Naturally, these are shaped by power relations surrounding who can participate within them. Power relations adapt and change as these spaces are realised, with power gained from one space through skills, capacity and experience, used to enter and affect other spaces [24]. Places for power include household, local, national and global arenas. Veneklasen’s expressions of power (2002) [2] was helpful in identifying the four main expressions of power demonstrated within these places since devolution: power over; power to; power with and power within (see Table 2). The final aspect of power considered in the power cube is forms of power. This builds on ‘three dimensions of power’ work by Steven Lukes (1974) [25], and subsequent expansion by Veneklasen et al. (2002) to identify three main forms – visible, hidden and invisible [2]. Underlying the interfaces between actors, the dynamics at work within the power cube and the expressions of power lie individuals’ experiences of power and privilege, which shape their social exclusion (or inclusion) and access to the decision-making table [26].

**Table 2:** Forms of power and their expression in Kenya

Form of power	Definition (Veneklasen, 2002)	Expression in Kenya
Power over	Power is viewed as ‘zero-sum’ where the more power one person has than the less the other has. Having power involved taking it from someone else and then using it to dominate and prevent others from gaining it.	Power over was typically exerted by state actors at the top of the institutional hierarchy within the county, by the governor, the county executive committee member for health and the members of county assembly, with some county executive committee members for health adopting an authoritarian approach, limiting sharing of knowledge and information. Power over was demonstrated in interactions between state actors (members of county assembly and governor and county executive committee members). Power over was demonstrated by state actors (e.g. county executive committee member for health) and other providers, with some technical decision makers feeling unable to challenge decisions which may not be suitable. *“Well just to be frank with you sometimes it is not easy for us to say no okay [to politicians]… my seniors here are politically appointed so at any time now they can change in the cabinet level of the county… sometimes is difficult for us to stand up and change this.”* County Health Respondent IDI, Male45 Patronage norms led to misuse of power in some settings, with resources channelled to voters/ citizens from similar tribe as the more ‘powerful’ leaders. Community members were not informed of all the choices available to them, or of the benefits and disadvantages of those choices limited their access to knowledge.
Power with	Based on mutual support and collaboration to build collective strength. It helps build bridges and promote more equitable relations	Mechanisms for power with have been introduced according to the Constitution, e.g. public participation meetings. However, failure to address norms which limit power within e.g. patriarchal norms, have led to limited active participation from many citizens, leaving these forums open to elite capture and limiting opportunities for power with. Overall, most county level state actors have made limited attempts to share priority-setting power with actors at other levels. *“The issue of health care in my view is no more in the hands of the health care providers, but rather in the [hands of] policy makers*.” County Health Respondent IDI, Male24 Exceptions include: one county where county level actors have plans for broader decentralisation to lower levels; in another county the county executive committee sought to reduce the power imbalance by sharing knowledge with actors from community level to county level, finding common ground and understanding among the interests of actors from all levels.
Power to act	Refers to the potential of every person to shape their life.	Outside of county level technical and political actors, other potential decision makers such as health workers, sub-county actors and community members appear to have limited power to act, with limited meaningful participation. *“So in a way we are feeling there is a gap. It [decision-making] is happening at the county level but they are not involving the most important people.****.****. We are not involved in decision-making nowadays in the county government.”* CHEW IDI, Male01
Power within	Relates to a person’s sense of self-worth, values and self-knowledge, having the capacity to have hope and affirming dignity and fulfilment.	Power within relates closely with how forces and structures, such as patriarchy and patronage remain unaddressed and as a consequence there has been limited scope for empowerment and increasing citizens power within to enable them to fully engage with priority-setting (see power with above). *“…members of county assembly are not very comfortable with the system [public participation] because they believe it is empowering the citizen so much that they are losing the political grip and that has been the issue across the country.”* National Respondent IDI, Male10

### Methods, participants and process

We conducted individual interviews with 269 individuals and 14 focus group discussions with a further 146 participants (see Table 4 [16]) between March 2015 – April 2016. Participants include 14 purposively selected national level key informants with specialist knowledge of the health priority-setting process. We purposively selected 120 county level decision-makers from ten diverse study counties (see Tables 3 and 4 [16]) to include a range of actors involved with priority-setting, including: politicians involved with decision-making for health, county treasury staff, gender and children’s office representatives and technical decision makers for health including members of the county health management team. We continued to interview across counties, due to the diversity of contexts and continued until saturation was reached by respondents at county level. Saturation was considered reached when there were no new major themes emerging from the findings. In-depth interviews (IDIs) with 49 health workers from sub-county, health facility and community levels were carried out in three (out of the ten) counties. Due to research time and resource constraints it was not possible to conduct this depth of research across all ten counties studied for the county level interviews, however, we sought to ensure as much diversity of responses as possible, by including counties which represented urban, rural agrarian and rural pastoralist settings.,We carried out interviews with 86 close-to-community (CTC) providers, their supervisors and community members and 14 focus group discussions with community members from two counties (out of the three) (see Table 4 [16]). This data was collected as part of an ongoing REACHOUT CTC provider quality improvement study in two counties (urban and rural agrarian). REACHOUT is an ambitious five year international research consortium aiming to generate knowledge to strengthen the performance of CHWs and other close-to-community (CTC) providers in promotional, preventive and curative primary health services in six low- and middle-income countries in rural and urban areas in Africa and Asia, including Kenya.

A primary point of contact (usually the county executive committee member for health) in each of the ten counties was approached to introduce the study, either through email or face-to-face introduction. This point of contact then typically provided introduction of the interviewer to other study participants. Participants were interviewed at a location convenient to them which afforded privacy, typically at their place of work. Qualitative data were recorded with participant’s consent and transcribed verbatim by research assistants with extensive transcription experience, supplemented by note taking by the researcher who conducted the interviews. Interviews typically lasted 40 to 60 min. We used topic guides, with questions which explored the actors and priority-setting processes for health following devolution; participants understanding of the implications of priorities set, particularly for access, use and effective coverage of health services. The topic guides were developed through an iterative process following informal discussions with national key informants and colleagues and a period of reflection and revision after data collection in one county to ensure questions elicited the responses sought.

National, county and some health worker level interviews were carried out by a foreign researcher in English language (RM), who completed the research as part of doctoral studies and who has received training in qualitative data collection and analysis. RM had no prior relationship with any of the research participants. Community and some health facility level respondents were interviewed by trained research assistants in Kiswahili or Kamba (depending on respondents’ preference).

### Analytical process

We adopted a framework approach to analysis in order to classify and organise data according to the key themes, concepts and emerging categories [27]. This included an inductive aspect, which allowed meaning to emerge from the data [28] through familiarisation with the data by reading and re-reading transcripts. Following this a coding framework was developed which drew on understanding of the literature, the objectives of the interview, the themes within the data collection tool and issues raised by the respondents themselves during interviews. Nvivo 10 software was utilised to manage and code data. Following coding, data was charted in order to summarise data while still retaining its context and essence [27], based on data from all ten counties. Data coding and preliminary analysis was carried out by RM, with regular discussions with LO, MT, TM and ST through the data collection and analysis process. Preliminary findings were presented to fellow researchers (including SM) engaged in health systems research in Kenya at an early stage in the data collection process, allowing the opportunity to develop data collection and analysis plans. Further presentation of findings with NM, LO, MT, TM, ST provided opportunities to critique and refine the analysis. SM and EB participate in a health systems governance learning site, conducting related research within Kilifi county in Kenya. Findings were then analysed collectively, highlighting differences between counties or types of respondent where appropriate. Given the nature of the themes which emerged from the findings, there was a strong fit between these themes and with Gaventa’s power cube and Veneklassen’s expressions of power (2002). We therefore carried out a re-analysis in light of these themes, given the similarity between our findings and these frameworks, in order to further the nuance of the analysis and to enable a deeper look at power in all its manifestations. Final analysis and manuscript development were finalised with inputs from all authors.

### Trustworthiness and ethics

Discussions and interviews conducted in Kiswahili or Kamba were translated to English, with a selection back-translated for quality checking. Data was triangulated between sources to minimise bias. We reflected on our position as UK and Kenyan researchers and adopted reflexivity and positionality lenses within the analysis approach. Regular discussions and presentations with colleagues and other researchers within and outside Kenya were an important part of maintaining validity throughout the research process. All participants were provided with information about the nature of the study and it’s objectives and gave informed written consent. Ethical approval was received from Kenya Medical Research Institute (KEMRI) and Liverpool School of Tropical Medicine, with research permit from National Commission for Science Technology and Innovation (NACOSTI).

## Results

Our results relating to power are presented in line with Gaventa’s power cube and Veneklassen’s expressions of power, as these are a natural fit according to the power-related findings arising from the data.

1. Place and expression of power

Global - Literature describes that devolution in Kenya was influenced by international pressure placed on the Kenyan government to finalise a new Constitution and introduce devolution reforms, as part of the National Peace Accord (2008) in the wake of 2007’s politically instigated violence following the controversial general elections [29].

National and sub-national - Since devolution, power dynamics and relationships have changed dramatically following the creation of the 47 new county governments (see Table 2 [16]). Nationwide restructuring resulted in the establishment of two levels of government (national and county). The former provincial level was removed, a new county level was created and authorities and responsibilities for the national and district (sub-county) levels have changed.

*“Decentralization has only recognized two layers of government; the national level and the county level, so now resources are only allocated to counties.”* National Respondent IDI, Male11

National - Respondents described how those working at the national level saw a decline in their power to determine priorities or assign budget at sub-national levels, compared with prior to devolution (see Table 2 [16]).

Sub-national - County level governments took on responsibility and power for planning, budgeting and implementing health services from community to county referral hospital level. The ability to influence health priorities within the county was typically viewed as primarily remaining in the grasp of state actors (the governor, the county executive committee members and members of the county assembly). Health managers were involved to varying degrees within the process, but were not typically viewed as key decision-makers.

*“The issue of health care in my view is no more in the hands of the health care providers, but rather in the [hands of] policy makers*.” County Health Respondent IDI, Male24

Health worker – Health workers, particularly at sub-county, health facility and community levels often described feeling excluded from both county and community decision-making structures.

*“So in a way we are feeling there is a gap. It [decision-making] is happening at the county level but they are not involving the most important people... We are not involved in decision-making nowadays in the name of system of the county government.”* Kitui County CHEW, Male01

Local – Pre-existing avenues for community participation exist, including community representation within quarterly meetings, health facility management committees, participation during community dialogue days. In addition, new avenues such as public participation meetings, where community members including representatives for women, youth and people with disabilities [14], have been introduced to provide a forum for community to participate in selecting priorities for health (and other sectors). However, community members who participated in our study did not describe participation in decision-making as one of their roles regarding health. In part this may be due to limited efforts from county actors to encourage attendance and active involvement of community members, including ‘vulnerable groups’ (see [21]). In addition, the education of community members to understand their role in decision-making was a positive exception, rather than the norm (see box 2 [16]).

The key actors are largely operating in new territory (see introduction). To further complicate this there is lack of formal guidance and clarity about roles and responsibilities for priority-setting. Leaders therefore need to negotiate for power alongside the roll-out and enforcement of priority-setting and service implementation. Political influence, control of resources and knowledge were viewed as important sources of power and are used by key decision-makers to exert and extend their ‘power over’ other actors (see Table 2). ‘Power over’ was by far the most common expression of power described by respondents within priority-setting for health at county level, although all four forms are present to varying degrees (see Table 2). Our study identified that negotiations surrounding power has created five main interfaces between actors within each county (see Fig. 2, modified [30]). These are explained more in the following section.

1. Between state actors (CEC and county assembly) – these negotiations tended to demonstrate attempts to exert ‘power over’ the other state actor for reasons of political gain.
2. Between state actors (CEC and county assembly) and providers (county level technical decision makers and health workers) - providers often felt that following devolution, power for priority-setting had relocated from health service providers to state actors.
3. Between providers (county level technical decision makers, sub-county managers and health workers) - providers working at levels below county level (sub-county, health facility and community level) often felt excluded from the priority-setting process, with limited scope for ‘power with’ other decision-making actors.
4. Between state actors (CEC and county assembly) and the community - negotiations between state actors and the community ought to be stronger following devolution, with the introduction of public participation meetings. Challenges exist however, with these mechanisms failing to address underlying norms or promote ‘power within’, thereby creating a barrier to empowering the whole community with ‘power to’ identify priorities (see section 3 community accountability and empowerment [21]).

Between providers (health workers) and the community - health workers at times felt excluded from county level decision-making structures as well as community level public participation meetings.

2. Spaces of power

Closed spaces: Our findings reveal that elites (those persons who hold access to the control of power and resources) often make decisions behind closed doors, this includes discussions between CEC members and the governor or among members of county assembly. We found that the degree to which decisions made occurred in closed spaces appeared to be influenced by the leadership style of those leading the priority-setting process. The CEC member for health was most frequently described as leading the priority-setting process for health and playing a key role in negotiating between decision-making actors when priorities differed. As a result, in a county where the CEC member for health has an authoritarian leadership style, it is likely that the priority-setting process will be less participatory, compared with a leader who recognises the value of other actors’ contributions. In our study, respondents from three out of ten counties described a CEC member for health (either present or recent past) who adopted an authoritarian or dictatorial approach to decision-making, with staff feeling disrespected and demotivated as a consequence. In these counties other members of the county health management team had limited knowledge about what, how or why decisions were made. Within counties with a leader who adopted an authoritarian leadership style, even those involved with discussions at times felt unable to question or challenge priorities without fear of reprisal.

*“I also think there is the power balance. There [are] also some powerful positions …yet the decisions you (the person in power) make are not good, and I am below you and I know, we are supposed to do Y, yet you are saying we should do X and because of that power relations there is no way I am communicating with you without the fear of losing my job, then we make the wrong decisions.”* County Health Respondent IDI, Female69

Respondents from three counties did not describe particularly strong examples of either authoritarian or participatory leadership approaches. Meanwhile, respondents from four counties described the use of participatory approaches to priority-setting, opening up the priority-setting space to include other stakeholders across the health system. In the clearest example of this within a positive deviant county which demonstrated strong participatory decision-making approaches, a CEC member sought to ensure that other actors within the county health management team, the county assembly, health workers, administrators, community health workers and community members were all empowered with knowledge about health and health priorities and were actively involved in identifying priorities, informed by this knowledge.

*“The process of making decisions is not a one man show. I would like to say that first of all, even the strategies that we have developed, there is what we call public participation every year we ask the public what are priorities they have? What do they need? … and we do also have community representatives during the budgeting process so that it is all inclusive so that whatever we are doing is what the people want.”* County Health Respondent IDI, Male 50

Invited space: Citizens are invited to participate in priority-setting by authorities, which in Kenya includes the public participation forums (see [16] for more details about the public participation forums, barriers to meaningful engagement and examples of best practices). These public meetings have been introduced since devolution as a forum for accountability, with the objective of ensuring community needs and priorities are reflected in county government plans and budgets. Ideally, the meetings should promote a ‘power with’ approach to priority-setting, providing a range of actors with the knowledge and opportunity to take part in actively selecting priorities and monitoring progress towards their attainment. However, failure to address underlying social norms and structures contribute to limited ‘power within’ by ‘ordinary’ citizens, including those most marginalised (see Table 2). Community members from formerly marginalised counties in Northern Kenya are reluctant to participate in meetings held by government actors to identify their needs and priorities, due to their perception of unchanged historic norms arising from colonisation which led to decades of neglect within their region.

*“…they (community) still have the perception that this is the government that has neglected them for all those years …actually a lot civic education needs to be done if you want actually effective participation.”* County Health Respondent IDI, Male48

As the quote indicates, for these populations and other marginalised groups (such as women, youth, street dwellers), historic, social, cultural norms and discriminations continue to persist (despite legislation which provides scope for their participation). As a consequence these norms impose limits to citizens’ sense of self-worth, self-knowledge and ‘power within’, thereby limiting their agency to take up their ‘power to act’ now made available according to policy in the invited space of public participation forums (see Table 2). In general, there have been limited efforts made to educate community members to understand their roles and responsibilities for participating in decision-making, leading to continued knowledge imbalance, and limiting the scope for genuine community empowerment and participation within priority-setting processes. This is further explored through the ‘invisible’ forms of power operating, which influence an individual’s agency to move into invited spaces (see Table 2).

In fact, not only are marginalised community members reluctant to participate due to limited ‘power within’, but there was suggestion by a minority of national level respondents that there may be reluctance on the part of county leaders to share power with the community in general, due to the perception that by empowering the community they would themselves lose power. As a consequence these invited spaces became tokenistic, failing to promote the ‘power with’ approach to priority-setting between county level and community actors as intended.

*“A lot of other counties are actually hesitant to implement public participation models because they want to have all the power, not to give the communities power.”* National Respondent IDI, Male10

Claimed spaces: These are spaces claimed by less powerful actors from existing power holders. These were infrequently described by respondents in our study. There was little discussion about how health workers can engage and re-claim power from politicians to ensure their involvement and technical appropriateness of priorities set. Although the recurring health worker strikes following devolution may be considered to indicate their efforts to re-claim power, as they pressed for re-centralisation of health service delivery to national government [31]. A minority of respondents acknowledged a gap for citizens to hold their service providers to account. In response to this gap, there were some early examples of social media platforms being established by citizens to raise awareness about poor quality services.

*“…currently sometimes even in the internet [like] facebook, what you usually see [is] people writing things. So you realize that sometimes, something that should have been solved earlier, blows up into a political matter…It should have been very simple, because if you had gotten in touch with the person who was complaining…But we don’t have a system even of measuring the satisfaction of the people.”* County Health Respondent IDI, Male09

Other respondents, particularly county level technical respondents, highlighted the need for citizens to hold leaders to account for providing (or not) health services. However, some citizens themselves highlighted their confusion surrounding how this could be done.

3. Forms and visibility of power

‘Visible power’ - Within Kenya there is a clear general process for how priorities should be set, which is outlined within the new Constitution, although it contains limited specific guidance for how the process should be implemented. In many instances respondents described examples where visible power was used to tackle inequities, with county governments seeking to address inequitable availability and access to health services, through investment in infrastructure and recruitment of additional human resources for health. In general this process was followed across the study counties. However, while the process was followed, some of the steps were merely perfunctory in nature – fulfilling the requirements but not striving towards attaining the objectives specified in the Constitution.

*“It [decision-making process] is just but it is not fair (laughter) …It is just because the process is followed and people are involved in the decision-making. But it is not fair, because you go through that process and at the end of the day, you don’t achieve anything.”* County Non-Health Respondent IDI, Female34

‘Hidden power’ –As a consequence of the rapid roll-out of devolution, additional guidance surrounding priority-setting processes and structures (including accountability mechanisms), county laws and policies and recruitment of needed staff had not yet been put in place when the reforms started. This created a vacuum at county level, which allowed ‘hidden power’ to have a disproportionate effect, with certain powerful actors able to control the extent to which other actors are included (or not) in identifying priorities, the content of the priority-setting agenda and ultimately the priorities set. In our study many community members and providers (including technical health systems decision-makers from county, sub-county and health facility level) highlighted that tribalism and patronage norms (which were common within the former centralised government), have continued in some counties under the devolved government.

Politicians, including the governor and members of county assembly, were commonly perceived by county technical decision-makers and national key informants to be motivated to a large degree by their own political aspirations, desire to secure votes during election or to repay promises made as a result of patronage. This was felt to have led to preference and over-investment in visible and popular priorities such as ambulances and infrastructure over less visible public health interventions or quality improvement, with more resources distributed within areas of political support for a powerful leader, rather than the area of greatest need.

*“So as a governor, if these are my voters in terms of my investment I would rather focus on these pockets that give me votes, rather than these ones who still need a lot of support in health.”* National respondent IDI, Male05

‘Invisible power’ – In our study, community members themselves did not generally consider priority-setting as one of their health-related roles. Underlying norms and discriminations such as tribalism, sexism, classism or ableism have not been adequately addressed since devolution, with inadequate actions to accommodate attendance at community meetings by ‘marginalised’ groups. This is despite measures having been introduced in policy including guidance specifying that no more than two thirds of membership of county assembly are of the same gender, 5 % of community representatives appointed or elected to public bodies are persons with disability, and that the state should put in place affirmative action programmes to ensure minorities and marginalised groups participate and are represented in governance [14]. According to the Constitution, those identified as marginalised include women, persons with disabilities, youth, ethnic and other minorities and marginalised communities (small/ traditional/ indigenous community, pastoralists) [14]. Even more worrying is the absence of any guidance within the Constitution for other marginalised groups, including gay and lesbian persons, street dwellers, drug and alcohol users or extreme poor. All these groups were identified (to varying degrees) as ‘vulnerable’ by study respondents, yet there is no clear legislation to promote their participation in decision-making. Unsurprisingly, power differentials between marginalised groups, other community members, politicians and actors facilitating meetings persists. Our findings reveal that in patriarchal communities many women may have too many responsibilities at home to be able to attend public participation meetings or do not have the confidence to speak out even if they do attend. Those who were illiterate, poor, have a disability, live far from the meeting location or don’t read English (the language meetings are advertised in) experienced barriers to attending and/or participating. For such reasons, power imbalances permit the views of more powerful actors to dominate leaving priority-setting open to manipulation by local elites, which can lead to selection of inappropriate priorities.

*“…some of the challenges are political because you realise politics plays in almost everything so if a person wants something you just go to the community and pressure them and these people the community come up with a wrong decision on health because of politics … based on someone else’s interest which is not good.”* County Non-Health Respondent IDI, Male33

## Discussion

We found Kenya’s transition towards devolution is transforming the former centralised balance of power, leading to greater ability for influence at the county level, with reducing power at national and sub-county (district) levels and limited change at community level. Within these changing power structures, politicians are felt to play a greater role in priority-setting for health. The interfaces and tensions between state politicians, technical health service providers and the community has at times been felt to undermine technical priorities. While power has changed drastically at the higher levels, there has been varied and typically limited consistent change at community level. Underlying social structures and discriminations generally continue unchanged, leading to the continued exclusion of those most vulnerable from priority-setting processes. Through application of Gaventa’s power cube framework and Veneklasen’s expressions of power, we systematically identified the changing power distribution since Kenya devolved services in 2013 and the influence of social norms, structures and discriminations on the distribution of power, the space of power and the visibility of power.

Devolution in Kenya has reduced the power formerly enjoyed by national authorities. Political actors at the county level now have unprecedented ability to set priorities and to control resources, leading to increased power at this level. Politicians are often motivated to provide electorally appealing services which will consolidate political support and maximise their voter base in pursuit of re-election [32]. The politicisation of priority-setting for health can bring positive results towards improving access to health services, with UHC having previously been recognised as an ‘electoral asset’ [33]. However, there are also threats that local patronage will influence the provision of health services, contributing to an accumulation of services within home areas for more powerful decision-making actors [21]. In keeping with findings from Philippines, Indonesia and Kenya [11, 18, 34], health workers from sub-county and facility level in Kenya have experienced a loss of power. In keeping with an earlier study in Kenya, we found that these health workers described exclusion from the county-level priority-setting processes and a lack of clarity surrounding their new role following introduction of devolution [18]. This has at times led to the setting of priorities which do not always meet urgent technical needs, contributing to demotivation among health workers. An earlier study in Kenya found that sub-county level managers had sought to ‘claim power’ and support other health workers, within the space available to them [18]. At community level, mechanisms to engage citizens within priority-setting have been established, but as the Additional file 1 by McCollum et al. (2018) highlights, these mechanisms face major constraints to facilitating the meaningful participation of ‘ordinary’ citizens [16]. These constraints largely reflect those identified by Cleary et al.(2013) [35] and include limited respect from certain actors, limited investment and allocation of resources to facilitate involvement of marginalised groups and failure to address underlying values, beliefs and cultures which act to exclude certain actors from the decision-making table.

Objectives for devolution in Kenya (such as citizens’ empowerment, promotion of national unity and eradication of inequalities [14]) are in part an effort to alleviate the effects of structural forces such as colonisation, which had led to the ethnocentrism fuelled violence during 2007 elections and long-standing inequities based on geographic location or tribe [29]. Local, regional, national and international norms, forces and systems form a complex web that combines to create and sustain social and health inequities [36]. In keeping with aspects of two recent Kenyan studies [37, 38] our findings reveal that patronage norms, including former politicians’ encouragement of citizens to evaluate their leader based on his/her ability to fund projects for certain groups in local communities [38] have not yet been eradicated. Instead, in a minority of counties these norms have found ways to flourish within the devolved governance system, leading to nepotism and service distribution which aligns with patronage networks rather than need. This has contributed to over-emphasis on visible curative services, to the neglect of less tangible but often more equitable and cost-effective public health services, such as community health. This exploitation of the priority-setting process to secure votes is in keeping with the ‘majority voting model’ outlined by Goddard et al. (2006) [32] and previously observed in Tanzania [39]. Respondents from formerly marginalised counties spoke about the reluctance of citizens to engage with community governance mechanisms, highlighting the need to first address the political and historical legacies before citizens will participate [40]. In general, community members do not seem to have been adequately informed about their role within priority-setting, the choice of interventions available to them or provided with the data which would allow them to make informed decisions. This is similar to other contexts where governments obstruct or resist community participation, which raises concerns about living conditions or proposes solutions [39, 40].

In Kenya, and other contexts undergoing health systems change there is need to strengthen meaningful community empowerment, by addressing underlying norms and structures which create a barrier to the expression of ‘power within’ by disadvantaged community members and which encourages ‘power with’ and ‘power to’ expressions of power [2]. This will need specific actions to ensure inclusion of ‘disadvantaged groups’, standards placed on local governments and creation of conditions for deliberation within public participation forums [41]. Properly functioning accountability mechanisms should support governance and ensure answerability between actors [35] involved with setting priorities, providing services and their recipients within communities.

### Limitations

The diversity between Kenya’s 47 counties may limit generalizability of findings. The selection of ten study counties tried to ensure diversity in demographic, geographic, social, cultural and economic differences. Interviews were conducted with county leaders across ten counties, and with health workers in three and community members in two counties due to time and resource constraints, and so findings are not necessarily generalisable across the country. The restructuring and implications for power within priority-setting and at community level will adapt over time. As such, we can only ever present a snapshot of this in a particular time and place. However, we have tried to consider how historical factors have changed over time leading up to devolution and their impact for its implications. Positionality of the main interviewer as a foreign researcher may have inhibited some respondents from openly sharing their opinions. Conversely, some respondents may have felt less threatened and discussed more. Inclusion of experienced Kenyan co-authors in study design and analysis sought to bring ‘insider’ perspectives to the study.

### Recommendations

Power analysis of priority-setting at county level, after devolution in Kenya highlights the need for stronger institutional structures, processes and norms to reduce the power imbalances between decision-making actors. Potential opportunities to address these imbalances include:

1. Promote transparency and accountability between political and technical actors, by involving politicians (e.g. representative for health for county assembly) within routine quarterly planning meetings, allowing opportunity for technical actors to share routine monitoring, progress, challenges faced and to respond to any questions or concerns from politicians.
2. Build capacity of political actors to understand health holistically, including preventive, promotive, curative and rehabilitative services. Build capacity of health providers to understand the needs of politicians so as to enable them to present politicians with easy to interpret information about cost-effectiveness, effectiveness and equity aspects of various health interventions. Provide opportunities for politicians to interact with and hear from community health volunteers to understand more about their work.
3. Build stronger platforms to engage health workers at different levels (community and health facility level) in priority-setting, in order to build ‘power within’ and encourage their participation and sharing of ‘power with’ other actors in priority-setting. Along with promoting transparency between health service providers and state actors.
4. Ensure governance and accountability measures, such as public participation are meaningful, by reducing the knowledge imbalance (between the community and other technical and political actors) to develop ‘power within’ and encourage ‘power to’ participate in priority-setting by different community members. Provide community members with easily understood information about the range of choices available to them, the reasoning behind those choices and the process for filtering choices. Create innovative approaches to ensure participation from those considered ‘marginalised’ in priority-setting, such as women only meetings in certain contexts or use of social media platforms with youth.

## Additional file

Additional file 1: **Appendix 1.** Topic guides. (DOCX 21 kb)

## Abbreviations

CEC: County Executive Committee
CTC: Close-to-community
FGD: Focus Groups Discussion
IDI: In Depth Interview
