# Supplementary material for: “Sometimes it is difficult for us to stand up and change this”: an analysis of power within priority-setting for health following devolution in Kenya
Source: BMC Health Serv Res. 2018 Nov 29;18:906. doi: 10.1186/s12913-018-3706-5 (PMC6264027; doi:10.1186/s12913-018-3706-5)
Supplement: Supplementary file 1 — Appendix 1. Topic guides. (DOCX 21 kb) [file 12913_2018_3706_MOESM1_ESM.docx]

## **Priority-setting, Devolution and Community Health Topic Guide**

*Many thanks for agreeing to the interview, I would like to start by asking you about how decisions are made and priorities set in this county*

**Priority-setting**

1. Please tell me more about your role, relating to health within the county
2. Who are the main people involved with making decisions/setting priorities about health services in this county
3. What are the health priorities specific for this county?
4. How are priorities set for health in this county?
5. How do you make decisions about service provision for health priorities in the district?

**Relevance/criteria**

1. What kind of information is used for priority-setting?
2. What factors/criteria/values are taken into account when setting priorities?
3. In your opinion do you think the process is relevant to the needs and challenges of your county?

**Implementation**

1. Please describe how your priorities are implemented

**Communication**

1. What happens after a decision is made?
2. How do you disseminate the priorities set?
3. Do you think that the process you have described is publicly accessible?
4. How do you communicate with the recipients of priorities?

**Equity**

1. What is your understanding of equity/fairness?
2. Do you think that the process for setting priorities is fair/equitable? Please explain

**Accountability**

1. What is your understanding of accountability?
2. Do you think that the process for setting priorities is accountable? Please explain

**Transparency**

1. What do you understand by transparency?
2. In your opinion do you think the process is transparent?

**Appeals and revision**

1. Please tell me about opportunities for appeal and revision within the process?
2. How do you resolve disagreements on a priority?

**Leadership**

1. Who leads the process of priority-setting?
2. Tell me more about leader’s initiatives to ensure implementation of the priorities identified?
3. How would you describe the skills of leadership?

*Now I would like to ask you more about devolution*

**Devolution**

1. What do you think is the purpose of devolution for health services?
2. What are the main changes which you have seen since devolution?
3. What changes have you seen in decision-making for financing and setting budgets?
4. What changes have you seen in decision-making for service delivery?
5. What changes have you seen in disease surveillance and response to disease outbreaks?
6. What changes have you seen in decision-making for human resources?
7. What changes have you seen in supply chain for drugs and supplies?
8. What changes have you seen for equipment and transport?
9. What changes have you seen for infrastructure?
10. What changes have you seen for health and management information systems?
11. What changes have you seen in decision-making for governance and community participation?
12. What is the most significant change? Why?
13. What are the benefits and challenges of these changes?
14. What indicators do you think should be used to measure progress or performance for health since devolution?
15. Has how you set priorities changed since devolution? Please explain
16. Do you feel you have the space to make all the decisions needed within the county? Please explain
17. Who are vulnerable groups?
18. Has how you provide services for vulnerable groups changed since devolution?
19. Is health and service provision for vulnerable groups being tracked through devolution? Please explain

*Now I would like to discuss more about the community health strategy*

**CHS progress to date**

1. Please tell me about how the community health strategy has been rolled out (in this county)?

**CHS indicators**

1. What do you think are the most important indicators to measure performance of community health?

**Decision-making for community health**

1. What are the mechanisms for CHWs and CHEWs to feed into policy and practice?
2. How are decisions made about where community units are established?
3. Do you feel that devolution has influenced provision of community health services?
4. What would you say is the ratio for allocation of the current budget for curative vs preventative services within the county?
5. Is data from dialogue days used in making decisions for community health? How?

**CHS Equity**

1. Can you tell me how equitable you think the community health strategy is?
2. What would equitable community health services look like?
3. To what extent do you feel that community health services in this county addresses the needs of vulnerable people? Why? How?
4. Are there any other groups of people within the county who don’t benefit from community health services? Who are they? Why don’t they benefit?
5. Do you feel that CHWs are representative of the members of their community?

**CHS Sustainability**

1. Can you tell me more about how sustainable you feel the community health strategy is? Please explain
2. What is the county’s role in ensuring sustainability of the CHS? How can this be improved?

**Suggestions**

1. Do you have any additional suggestions about what else would help make the provision of community health services more equitable?

41. Is there anything else which we have not discussed but you feel is relevant to this topic?

## **Topic guide for health workers and community health personnel**

1. Please tell me more about your role and responsibilities in your current position
2. Please tell me what your role is for making decisions about health
3. Please tell me your feelings about devolution for health
4. Can you tell me more about what changes you have seen since devolution?
   1. What changes have you seen in how you make annual workplans and set your budget?
   2. What changes have you seen in how you manage finances and manage budgets?
   3. What changes have you seen in service delivery?
   4. What changes have you seen in disease surveillance and response to disease outbreaks?
   5. What changes have you seen in staff availability? Staff turnover? Staff performance? Supervision of staff?
   6. What changes have you seen in supply chain for drugs and supplies?
   7. What changes have you seen for equipment and transport?
   8. What changes have you seen for infrastructure?
   9. What changes have you seen for health and management information systems?
   10. What changes have you seen in governance, community participation and relationship to community?
   11. Do you feel that devolution has influenced provision of community health services?
   12. Probe Benefits and challenges
5. Who do you feel owns these changes for health?
6. Please tell me more about if/how your role has changed since devolution
7. What does equity mean to you?
8. Who do you think are vulnerable groups?
9. To what extent do you feel that the (community) health strategy addresses the needs of vulnerable people?
10. Have you seen any changes to how health services are provided for vulnerable groups since devolution?
11. To what extent does this sub-county/ health facility/community unit have flexibility in responding to local needs?
12. Do you receive feedback on decisions made and on your progress and equity of this?
13. Do you think that the current structure for decision-making during annual planning and budgeting for health is relevant to your needs and challenges? Why? Why not?

**Community health specific**

1. How are decisions made about where community units are established?
2. Please tell me more about the benefits devolution creates for community health
3. Please tell me more about the challenges devolution creates for community health
4. Any other comments on how to improve?

## **National Level Priority-setting, Devolution and Community Health Topic Guide**

**Priority-setting**

1. Please tell me more about your role, relating to health
2. What role do you/ your department play in guiding decision-making for community health/budget/ general health?
3. What have been the benefits/ challenges associated with your role?

**Devolution**

1. What do you think is the purpose of devolution for health services?
2. Do you feel that this purpose is being achieved? Why?
3. When was the handover of health service delivery from national to county governments made?
4. What are the main changes for health which you have seen since devolution across the country?
5. What changes have you seen in decision-making for financing and setting budgets?
6. What changes have you seen in decision-making for service delivery?
7. What changes have you seen in disease surveillance and response to disease outbreaks?
8. What changes have you seen for immunisation?
9. What changes have you seen in decision-making for human resources?
10. What changes have you seen in supply chain for drugs and supplies?
11. What changes have you seen for equipment and transport?
12. What changes have you seen for infrastructure?
13. What changes have you seen for health and management information systems?
14. What changes have you seen in decision-making for governance and community participation?
15. What changes have you seen for community health services?
16. What is the most significant change? Why?
17. What are the benefits and challenges of these changes?
18. What indicators do you think should be used to measure progress or performance for health since devolution?

**Relevance/criteria**

1. How should counties be setting priorities for health?
2. What kind of information should counties be using for priority-setting?
3. What guidelines are available to guide counties in decision-making?

**Implementation**

1. How effective do you feel implementation of health service delivery is following devolution?

**Communication and guidance**

1. What happens after guidance document is developed?

**Equity**

1. What is your understanding of equity/fairness?
2. Please tell me about policies which have been put in place to promote equity for health within Kenya
3. How effective do you think these policies have been in practice? Why?
4. What are the main challenges to health equity? Why?
5. Do you think that devolution is more or less fair/equitable? Please explain
6. How has devolution influenced health equity?
7. How can health equity be improved?

**Accountability**

1. What is your understanding of accountability?
2. Do you think that the process for setting priorities is accountable? Please explain

**Transparency**

1. What do you understand by transparency?
2. In your opinion do you think the process is transparent?

**CHS/ budget/ general advice**

1. Please tell me about how the community health strategy/ budgeting/ general health has been rolled out across the country (since devolution)?
2. What support/ guidance does national level provide for CHS/ budget/ general health?
3. Who is this support for?
4. How much money was allocated to CHS before devolution? Why?
5. How much money was allocated to CHS after devolution? Why?
6. How has community health changed since devolution? Why?
7. How has provision of CHS services changed since devolution?
8. Which counties have embraced CHS? Which have not?
9. What do you think are the most important indicators to measure performance of community health?
10. How are decisions made about where community units are established?

**CHS Sustainability**

1. Can you tell me more about how sustainable you feel the community health strategy is? Please explain
2. What is the national role in ensuring sustainability of the CHS? How can this be improved?

**Suggestions**

1. What are your current recommendations for county governments about community health/budget/ general health?
2. Is there anything else which we have not discussed but you feel is relevant to this topic?

## **Quality Improvement In-depth Interview Guide: Community Health Worker**

**Baseline Evaluation**

1. **Work Background**
2. Please tell us about your daily tasks and activities
3. What do you enjoy about your work as CHW?
4. How happy/ unhappy do you feel to do your work as a community health services provider?
5. How long/ how many years do you intend to work as a community health services provider?
6. What recognition do you receive for your work?
7. **Supervision**
   1. Please describe the supervision you receive.
   2. What do you feel about the supervision you receive?
   3. How do your different supervisors work together to coordinate your work within the program and outside the program?
   4. How could the supervision be improved to motivate you further?
8. **Referral**
   1. How do you conduct referral from the community to the facility?
   2. What are some of the reasons for community going or not going for referral?
   3. How do you think referral could be improved?
9. **Community Engagement**
10. How do you interact with the community in your work aside from visiting them at home?
11. What do you feel about how the community thinks about your work?
12. What is your role in enhancing community participation?
13. What challenges do you face in carrying out the role and what could be the reasons?
14. **Fairness in service provision**
    1. What does fairness in providing health services mean to you?
    2. Who are those people who find it difficult to seek health services in the community you work in? (Or which groups of people in the community you work in mostly need health services, but you face challenges in providing them with community health services)
    3. How are the people you have mentioned above able to get help for any community health needs they have (e.g. referrals, health education)?
    4. To what extent do you feel that you as a community health service provider meets the community health needs of these people within your catchment area?
    5. Is there anything that helps you to provide health services for everyone, including the people mentioned above in the community you work in?
    6. Are there any local initiatives that you are aware of which have been successful for ensuring that everyone in the community gets the community health services that they need?
    7. Do you have any ideas or suggestions about what would help ensure that everyone gets the community health services which they need?

## **Focus Group Discussion: Community level - English**

**BASELINE EVALUATION**

1. **Problem description**
2. What are the main challenges of getting health care in the community you live in?
3. What are the main challenges with health services provided by CHWs in the community you live in?
4. Who are there people in your community who don’t have regular contact with the CHWs? How come?
5. **Supervision of CHWs and CHEWs**
   1. What kind of supervision do CHWs receive?
   2. How do you think CHWs should be supervised?
   3. Who supervises the supervisors of CHWs?
6. **Referral**
7. In what circumstances do CHWs make referrals?
8. How do people feel if the CHW refers them to a health facility?
9. Whose advice do you trust or take when making decisions about seeking health care?
10. How do you think referral for health services should be improved?
11. **Community Engagement**
12. What role do you as community members play in the provision of community health services?
13. How is your participation as community members facilitated in the provision of community health services in your community?
14. What hinders you from participating in the provision of community health services in your community?
15. **Fairness in service provision**
    1. What does fairness in service provision mean to you?
    2. Who in the community you live in uses the services provided by the CHWs?
    3. Who are the people who face the most difficulties in seeking health services in the community you live in? (Or which groups of people in the community you work in mostly need health services, but community health service providers face challenges in providing them with services)
    4. How are the people you have mentioned above able to get help for any community health needs they have (e.g. referrals, health education)?
    5. To what extent do CHWs meet the health needs of these people within the community you live in?
    6. What challenges do you face in accessing and using community health services? Why do you think these challenges exist?

- 1. Has anything changed in the community you live in since the community health service providers started working there?
  2. What would make it easier for you and for vulnerable people in the community you live in to use community health services?
